# Of RNA-binding proteins and their targets: interaction determines expression

**DOI:** 10.1186/gb4155

**Published:** 2014-01-27

**Authors:** Bojan Zagrovic

**Affiliations:** 1 Department of Structural and Computational Biology, Max F. Perutz Laboratories, University of Vienna, Campus Vienna Biocenter 5, Vienna, A-1030, Austria

## Abstract

Combining the prediction of interactions between mRNAs and RNA-binding proteins with experimental expression profiles uncovers novel regulatory paradigms concerning proliferation and differentiation processes.

See related research, http://genomebiology.com/2014/15/1/R13

## RNA-binding proteins: computation comes to the rescue

RNA-binding proteins (RBPs) are the principal regulators of RNA metabolism [1]. From transcription, processing and stabilization to transport, storage and translation, all the main stages in the life cycle of an RNA are crucially dependent on interactions with different RBPs. Although experimental approaches are undoubtedly making impressive progress in mapping the landscape of RNA-protein interactions, only its main features have emerged thus far, and many specific details remain out of sight [1-3]. We have yet to identify a comprehensive set of all RBPs and are even further from arriving at a full catalog of their targets, let alone a mechanistic and functional characterization of the interactions between them. With these challenges in mind, in 2011 the Tartaglia group made available the catRAPID server, which was designed for facile, speedy prediction of interactions between RNAs and proteins based on physico-chemical principles [4]. Starting with secondary-structure information, hydrogen-bonding preferences and van der Waals forces, catRAPID not only predicts associations between RNA molecules and proteins from their primary sequences but also assigns interaction strengths and delineates binding sites. Initial validation efforts, as well as a growing body of subsequent research work, appear to strongly confirm the soundness of the predictions made by catRAPID and encourage its usage in studies of increasing ambition and scope [5,6], an inspiring example of which is presented in the current issue of *Genome Biology*[7].

## Regulation of constitutive gene expression patterns by RBPs

Tartaglia and colleagues aimed to address the difficult, yet tremendously important, question of how an alteration in RBP expression affects the abundance of RNA targets [7]. More specifically, the authors set out to probe the link between experimentally determined tissue-specific expression patterns of more than 1,000 known human mRNA-binding RBPs [2,3] and the equivalent patterns of thousands of mRNAs. The key ingredient to this analysis is provided by catRAPID and its predicted pairwise interaction propensities between members of the two sets. While these are experimentally known for just a small subset of molecules, the computational strategy has allowed the authors to cast a much wider net and address the problem at a proteomic scale.

Remarkably, the authors discover that mRNA-RBP pairs for which the catRAPID algorithm predicts a high interaction propensity tend to have strongly correlated or strongly anti-correlated expression patterns in the 14 human tissues examined. In other words, interaction between a given RBP and a given mRNA, as predicted by catRAPID, is with high statistical significance related to the probability that the two have linked patterns of experimentally determined expression levels. Importantly, putative interaction does not in any way foreshadow the direction of this linkage (correlated or anti-correlated patterns being equally represented), but it does foreshadow its presence. The fact that the same finding is observed regardless of whether one uses immunohistochemistry or RNA sequencing data to determine the RBP levels is intriguing, given that the two data types are not expected to overwhelmingly correlate with one another [8]. It is possible that the underlying phenomenon uncovered by Tartaglia and colleagues is so strong that it is robust to this discrepancy, shedding new light on the general problem of the relationship between the expression level of proteins and that of their transcripts.

## Functional relevance of linked mRNA-RBP expression patterns

What are the functional contexts for the reported strongly correlated and anti-correlated expression patterns of RBPs and their predicted mRNA targets? In order to address this question, Tartaglia and colleagues analyze the enrichment of Gene Ontology functional categories among the group of predicted interactors with highly correlated or highly anti-correlated expression patterns. Remarkably, they detect a strong enrichment of functions related to cell-cycle control among the positively correlated patterns and those for survival, growth and differentiation among negatively correlated patterns. What makes these results additionally interesting is the finding that over 90% of genes in both categories are listed in the annotated gene index of the Cancer Genome Anatomy Project run by the National Cancer Institute, with a large number of annotated tumor-suppressor genes featuring in the former category and many transcription regulators appearing in the latter category.

By interacting with their mRNA targets, RBPs can regulate protein expression at different points of the mRNA life cycle, ranging from transcription to translation to degradation. Therefore, in retrospect, it seems delightfully natural that the expression level of RBPs themselves would be crucial in regulating proliferative processes, including aberrant ones. Tartaglia and colleagues discuss a number of individual RBPs and mRNAs with particularly strong predicted interaction propensities, whose identification will be useful in designing new experiments.

## Significance and outlook

It is, in fact, precisely in this wealth of newly opened-up directions that the main strength of the paper by Targtaglia and colleagues resides. As a combined computational-experimental framework characterized by, on the one hand, specific individual hypotheses and, on the other, elegant, extremely relevant, large-scale implications, the study has high potential to guide and inspire future experimental work. Not all individual interaction predictions necessarily have to turn out to be true for this to be the case, but I for one am confident that the overall picture painted embodies important principles that are here to stay, robust to false discoveries in the prediction set.

When it comes to methodological details, a major contribution of the study is that it demonstrates the power of using expression profile data to discover novel regulation patterns at a global scale. Furthermore, the study successfully integrates computational predictions of RBP interactions with experimental expression profiles, showing that significant progress can be achieved even in the absence of definitive, experimentally determined interaction networks. Although computational predictions always require rigorous validation on a case-by-case basis, the overall strength of the correlations uncovered in this study, combined with the inherent simplicity of their potential biological rationales, further increase the confidence one has in the accuracy of catRAPID. In turn, this confidence further supports the idea that basic physicochemical principles, as embodied in the backbone of the algorithm, can provide a satisfactory foundation for understanding biological systems and processes as complicated as the regulation of cellular gene expression. Moreover, on this basis, computational predictions can lead to rich, experimentally testable hypotheses. In a related effort, we have recently analyzed the intrinsic propensity of individual amino acids to interact with different nucleobases, from which we suggest that proteins in general might exhibit a pronounced propensity to interact with their cognate mRNAs, especially if unstructured [9,10]. It is our strong belief that such a fundamentally hypothesis-driven, physicochemical paradigm will continue to prove fruitful in the future. We should therefore expect that many surprises of the kind delivered by Tartaglia and colleagues still await us.

## Abbreviations

catRAPID: fast predictions of RNA and protein interactions and domains at the Center for Genomic Regulation Barcelona, Catalonia; RBP: RNA-binding protein.

## Competing interests

The author declares that he has no competing interests.
